# Methods over Materials: The Need for Sport-Specific Equations to Accurately Predict Fat Mass Using Bioimpedance Analysis or Anthropometry

**DOI:** 10.3390/nu15020278

**Published:** 2023-01-05

**Authors:** Francesco Campa, Catarina N. Matias, Tatiana Moro, Giuseppe Cerullo, Andrea Casolo, Filipe J. Teixeira, Antonio Paoli

**Affiliations:** 1 Department of Biomedical Sciences, University of Padua, 35131 Padua, Italy; 2 CIDEFES—Research Center on Sports, Physical Education, Exercise and Health, Universidade Lusófona, 1749-024 Lisboa, Portugal; 3 Interdisciplinary Center for the Study of Human Performance (CIPER), Faculdade de Motricidade Humana, Universidade de Lisboa, Cruz-Quebrada, 1499-002 Lisboa, Portugal; 4 Fábrica da Pólvora de Barcarena, Atlântica-Instituto Universitário, Barcarena, 2730-036 Lisboa, Portugal

**Keywords:** BIA, body composition, futsal, body fat, predictive equations, skinfolds

## Abstract

Bioelectrical impedance analysis (BIA) and anthropometry are considered alternatives to well-established reference techniques for assessing body composition. In team sports, the percentage of fat mass (FM%) is one of the most informative parameters, and a wide range of predictive equations allow for its estimation through both BIA and anthropometry. Although it is not clear which of these two techniques is more accurate for estimating FM%, the choice of the predictive equation could be a determining factor. The present study aimed to examine the validity of BIA and anthropometry in estimating FM% with different predictive equations, using dual X-ray absorptiometry (DXA) as a reference, in a group of futsal players. A total of 67 high-level male futsal players (age 23.7 ± 5.4 years) underwent BIA, anthropometric measurements, and DXA scanning. Four generalized, four athletic, and two sport-specific predictive equations were used for estimating FM% from raw bioelectric and anthropometric parameters. DXA-derived FM% was used as a reference. BIA-based generalized equations overestimated FM% (ranging from 1.13 to 2.69%, *p* < 0.05), whereas anthropometry-based generalized equations underestimated FM% in the futsal players (ranging from −1.72 to −2.04%, *p* < 0.05). Compared to DXA, no mean bias (*p* > 0.05) was observed using the athletic and sport-specific equations. Sport-specific equations allowed for more accurate and precise FM% estimations than did athletic predictive equations, with no trend (ranging from r = −0.217 to 0.235, *p* > 0.05). Regardless of the instrument, the choice of the equation determines the validity in FM% prediction. In conclusion, BIA and anthropometry can be used interchangeably, allowing for valid FM% estimations, provided that athletic and sport-specific equations are applied.

## 1. Introduction

Assessing body composition is a widespread practice in the context of sports, and its quantification enables the monitoring of nutritional and health status among athletes [1,2]. Body composition characteristics also influence sports performance, since muscle mass contributes to force expression, whereas fat mass (FM) negatively affects aerobic power and movement patterns [3,4]. The percentage of FM is one of the most considered parameters in sports practice, and it can be measured through a wide range of techniques [5,6]. In this regard, the availability of user-friendly methods is crucial in the context of resource-limited settings such as team sports, where there is a need for low-cost, time efficient, and accurate assessment procedures [7].

Among the field tools developed to assess body composition, bioelectrical impedance analysis (BIA) and surface anthropometry represent two easy and feasible techniques that are frequently used in the context of sports, compared to more accurate but less available methods [2,8]. Particularly, the foot-to-hand BIA at 50 kHz and manual anthropometry performed using high-quality instruments have been proposed as valid tools when compared to less accessible reference techniques, such as underwater weighing, air plethysmography, and dual-energy x-ray absorptiometry (DXA) [8,9]. Despite recent advancements regarding accuracy using BIA and anthropometric procedures, a long-standing question [10,11] remains: which technique is more accurate between BIA and anthropometry to evaluate fat mass? Indeed, although this question has been open for decades, conflicting findings can be found in recent literature [12,13,14].

BIA and anthropometry allow for the estimation of parameters such as FM and fat-free mass (FFM) through predictive equations based on the relationship between the bioelectrical proprieties and the subcutaneous adipose tissue with body mass components [1,8]. Currently, a wide range of predictive equations are available in the literature, and each of these has been developed for specific populations, such as general and athletic subjects, as well as cohorts of athletes involved in different sports [8,9]. Therefore, the use of these formulas does not seem interchangeable, and their use should be carefully considered when evaluating body composition [15,16,17]. In previous sports-science related studies that compared BIA and anthropometry, not enough diligence was given to the choice of predictive equations [12,13,14], possibly resulting in conflicting findings over time.

Thus, the aim of this study was to clarify the comparative accuracy of BIA and anthropometry for assessing FM in the context of a specific sport, such as the evaluation of male futsal players. To accomplish this, different groups of predictive equations have been compared to DXA as a criterion method. This study hypothesized that the validity of the evaluation would depend on the assessment procedures, such predictive equations, rather than the instrument itself (e.g., BIA or anthropometry).

## 2. Materials and Methods

### 2.1. Participants and Study Design

A total of 67 futsal players competing in the Major Portuguese Futsal League “LIGA PLACARD,”(age 23.7 ± 5.4 years), were included in this cross-sectional study. All participants were ≥18 years old and did not take any medication or supplementation known to interfere with body composition assessment.

Generalized, athletic, and sport-specific BIA- and anthropometry predictive equations were selected according the following criteria:

Inclusion criteria:

- Published in an original article indexed on Scopus, PubMed, or WoS including healthy subjects, using high-quality instruments and standardized procedures [9,18,19].
- Anthropometric measurements obtained according to the International Society for the Advancement of Kinanthropometry (ISAK) protocol and included in the last ISAK manual [20].
- BIA performed using foot-to-hand technology at a 50 kHz frequency.
- Generalized predictive equations developed and validated on subjects from normal healthy populations.
- Athletic predictive equations developed and validated in samples including athletes from different sports.
- Sport-specific predictive equations developed and validated in futsal players.

Exclusion criteria:

- Dissertation or conference papers.
- Predictive equations developed for adolescents and elderly subjects.

When more than two predictive equations were identified for each group (generalized, athletic, and sport-specific) and method (BIA and anthropometry), only two of them were randomly selected using the random.org website. A total of four generalized [21,22,23,24], four athletic [25,26,27,28], and two sport-specific predictive equations [29,30] were selected and are reported in Table 1.

Data collection was conducted during the off-season. Informed written consent was obtained from all participants and ethical approval was provided by the Faculty of Human Kinetics Institutional Review Board (approval number 37/2021), attesting to the fulfilment of all human research standards set out by the Declaration of Helsinki.

### 2.2. Bioelectrical Impedance Analysis (BIA)

Whole-body BIA was performed using a single frequency of a 50 kHz device (BIA 101 BIVA^®^PRO, Akern Systems, Firenze, Italy), according to the guidelines for athletes [1]. The participants were instructed to remove all objects containing metal and to stay in a supine position during the measurements, isolated from the ground and electrical conductors, with legs abducted at 45°, shoulders abducted at 30° relative to the body midline, and hands pronated. After cleaning the skin with isotropyl alcohol, two intrinsic impedance adhesive electrodes (Biatrodes Akern Srl, Firenze, Italy) were applied on the surface of the right hand and two on the right foot. The accuracy of the BIA instrument was validated before each test session following the manufacturer’s instructions; the test– retest coefficient of variation in 10 participants for resistance (R) and reactance (Xc) was 0.4% and 0.2%, respectively.

### 2.3. Surface Anthropometry

Participants had their body mass and height measured to the nearest 0.1 kg and 0.1 cm, respectively, using a scale and a wall stadiometer (Seca, Hamburg, Germany). Skinfold thicknesses and girths were collected according to the selected predictive equations, with an accuracy of 0.1 mm and 0.1 cm, respectively, using a Harpenden skinfold calliper (Baty International, Burgess Hill, England) and an anthropometric measuring tape (CESCORF, Porto Alegre, Brazil). The technical error of measurement scores (TEM) was required to be within a 5% agreement for skinfolds and within 1% for girths [19]. Either the mean of the two measurements, or the median of the three measurements, was considered for analysis. All anthropometric measurements were performed by a level I-accredited anthropometrist, according to the standards of the ISAK [20]. Participants wore minimal clothing and no shoes during the assessment, which was conducted in a private environment. The anthropometrist’s test-retest coefficient of variation for the measurement of the same skinfolds and girths over 29 participants ranged between 0.10–2.24%.

### 2.4. Dual Energy X-ray Absorptiometry (DXA)

Participants underwent a whole-body DXA scan (Horizon Wi, Hologic, Waltham, MA, USA), according to procedures recommended by the manufacturer. The same technician positioned the patient, performed the scan, and executed the analyses in a ventilated room, with controlled temperature and humidity. The test-retest coefficient of variation for FM% in 29 participants was 1.7%.

### 2.5. Statistical Analysis

Data were analyzed with SPSS Statistics v.25.0.1.0, 2021 (IBM, Chicago, IL, USA) and MedCalc Statistical Software v.11.1.1.0, 2009 (Mariakerke, Belgium). All variables were assessed for normality with the Kolmogorov–Smirnov test. A paired sample t test was performed to compare the mean values obtained from DXA, BIA, and anthropometry. A linear regression analysis was performed, considering FM obtained from DXA method as the dependent variable and the estimated parameters as independent variables. Agreement was determined using the Bland–Altman method [31], Lin’s concordance correlation coefficient (CCC), including precision (ρ) and accuracy (Cb) indexes [32], and by McBride’s strength concordance [33] (almost perfect = >0.99; substantial = >0.95 to 0.99; moderate = 0.90–0.95; and poor = <0.90). Statistical significance was set at *p* < 0.05.

## 3. Results

**Figure 1:**
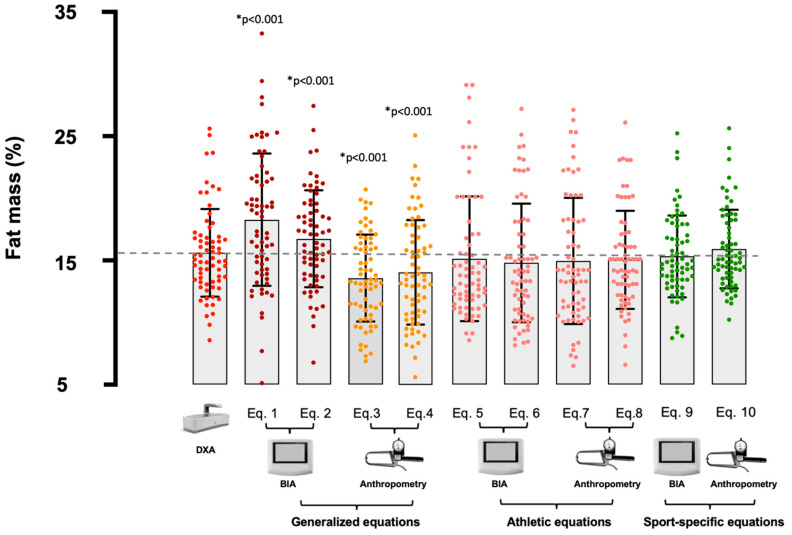
Mean and individual values for the percentage of fat mass (FM%) obtained from Dual X-ray Absorptiometry (DXA) and the selected equations; the upper and lower limits represent the standard deviation of the data. BIA = bioelectrical impedance analysis; Eq. 1 = Lukaski and Bolonchuk [21]; Eq. 2 = Sun et al. [22]; Eq. 3 = Durnin and Womersley [23]; Eq. 4 = Lean et al. [24]; Eq. 5 = Matias et al. [25]; Eq. 6 = Stewart et al. [26]; Eq. 7 = Evans et al. [27]; Eq. 8 = Witers et al. [28]; Eq. 9 = Matias et al. [29]; Eq. 10 = Giro et al. [30]. The dotted line identifies the mean value obtained with DXA; * = significant difference from DXA.

All generalized predictive equations showed a significant difference (*p* < 0.05) in mean FM% estimation as compared with DXA, as shown in Figure 1 and Table 2. The FM% estimated from athletic and sport-specific predictive equations showed no mean bias with respect to the DXA-derived FM% (Figure 1 and Table 2).

**Figure 2:**
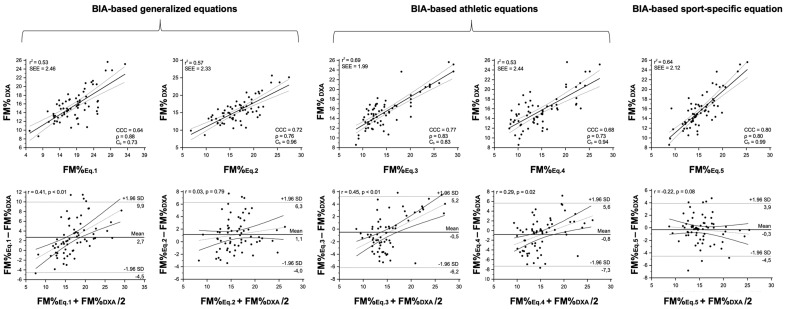
Results from regression, concordance, and agreement analyses between the percentage of fat mass (FM%) estimated from the selected BIA-based predictive equations and the reference method (DXA) in the futsal players. In the upper panel, the scatterplots show the relationship between the estimated and the reference FM% and the Lin’s concordance correlation coefficient (CCC), including precision (ρ) and accuracy (C_b_) indexes. In the lower panel, the results of Bland–Altman analyses are shown. Eq. 1 = Lukaski and Bolonchuk [21]; Eq. 2 = Sun et al. [22]; Eq. 3 = Matias et al. [25]; Eq. 4 = Stewart et al. [26]; Eq. 5 = Matias et al. [29].

**Figure 3:**
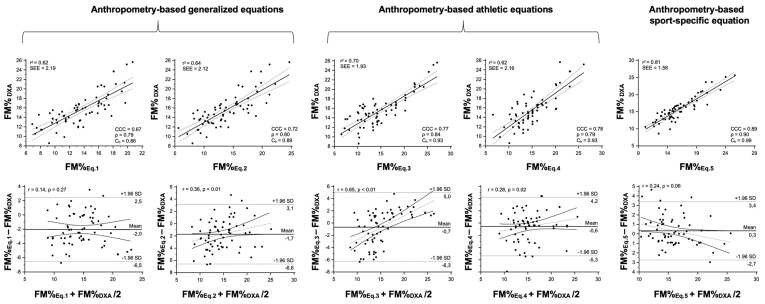
Results from regression, concordance, and agreement analyses between the percentage of fat mass (FM%) estimated from the selected anthropometry-based predictive equations and the reference method (DXA) in the futsal players. In the upper panel, the scatterplots show the relationship between the estimated and the reference FM% and the Lin’s concordance correlation coefficient (CCC), including precision (ρ) and accuracy (C_b_) indexes. In the lower panel, the results of the Bland–Altman analyses are shown. Eq. 1 = Durnin and Womersley [23]; Eq. 2 = Lean et al. [24]; Eq. 3 = Evans et al. [27]; Eq. 4; Witers et al. [28]; Eq. 5 = Giro et al. [30].

The selected athletic and sport-specific equations showed a coefficient of determination (r^2^) ranging from 0.53 to 0.69 and from 0.62 to 0.81 for BIA- and anthropometry-based predictive equations, respectively, as shown in Table 2, Figure 2 and Figure 3. Concerning the concordance analysis, the best performance was observed for the sport-specific predictive equation (Table 2, Figure 2 and Figure 3). In the agreement analysis, BIA- and anthropometry-based sport-specific predictive equations both reported no trend, whereas all the athletic predictive equations showed a positive and significant (*p* < 0.05) trend (Table 2, Figure 2 and Figure 3). Particularly, the sport-specific BIA- and anthropometry-based models showed a standard error of estimation (SEE) ranging from 1.58 to 2.12% (Table 2, Figure 2 and Figure 3). The CCC analysis (Table 2, Figure 2 and Figure 3) denoted a substantial strength of agreement and for the individual analysis concerning the Bland and Altman approach, no trend (*p*-value ranging from 0.057 to 0.083) was observed between the mean and the difference of the methods for FM% estimation (Table 2, Figure 2 and Figure 3).

## 4. Discussion

This study aimed to clarify whether BIA or anthropometry would lead to a better FM% estimation in a specific sport discipline. For this purpose, FM% estimated from generalized, athletic, and sport-specific predictive equations were compared with DXA-derived FM% in a group of male futsal players. Regardless of the instruments, the use of generalized equations led to poor FM% assessment in the futsal players, whereas sport-specific equations were more accurate than those developed using groups of athletes engaged in different disciplines. The present results show that when sport-specific predictive equations are applied, BIA and anthropometry can be used interchangeably, allowing for valid FM% estimations.

All generalized predictive equations showed a lack of agreement between the estimated FM% and DXA. However, BIA- and anthropometry-based equations showed heterogeneity in the direction of prediction: BIA-based predictive equations overestimated FM%, while anthropometry-based predictive equations underestimated FM%. These findings are in line with previous evidence [15] in which the use of generalized BIA-based formulas led to an underestimation of the FFM components and therefore, to an overestimation of the FM. The opposite result occurred when anthropometric formulas were used, and this could be explained by underlying theoretical bases. Anthropometry, and in particular, skinfold measurements, are based on the assessments of the subcutaneous adipose tissue thickness, which is directly related to the FM% [34]. Conversely, the relationship between bioelectric properties and body composition is based on the electrical conductivity of the tissues, typically of some FFM components [35]. Therefore, the aforementioned differences could explain discrepancies in FM prediction. Previous research did not always agree in identifying BIA as a valid tool for estimating FM% in a sports context, justifying that bioelectric properties are more informative for FFM-related variables, such as fluids or lean soft tissues [35]. Furthermore, previous evidence did not report similar findings, showing underestimation [36,37,38,39,40,41] or overestimation of FM% [42,43,44,45] with respect to the reference methods. This lack of agreement can be attributed to the different BIA technologies involved in these investigations, as well as to the poor choice of the predictive equations used [9]. A lack of consensus also exists in the current literature regarding the use of anthropometry-based generalized equations on groups of athletes. Some studies report that groups of generalized equations can be valid when applied in athletes [16,46], while others tend to display an underestimation of FM% [47,48]. In general, predictive regression models are accurate when applied to subjects with similar characteristics to those of the subjects involved in the studies used to develop those equations [49]. In this regard, athletic subjects should be considered to belong to a distinct population, as they present higher intracellular-to-extracellular water ratio, higher muscle mass, and generally lower levels of fat than the general population [50,51,52]. Considering the present results and the availability of specific equations for athletes in the literature, the use of generalized formulas to predict FM% in athletes should be discouraged.

In this study, both BIA and anthropometric assessment were valid when predictive equations for athletes were applied. The four selected athletic equations were developed for groups of athletes involved in different disciplines, including team, endurance, and velocity/power sports [43,44,45,46]. The present comparison showed a predicted FM% similar to that derived with DXA in the futsal players. However, when using the two sport-specific equations, it was possible to achieve greater accuracy and a substantial concordance level with both BIA and anthropometry. Although these findings suggest the importance of choosing the most appropriate predictive equation in estimating FM%, to date, few anthropometric-based [16]—and only one BIA-based sport-specific equation—are available in the literature [29]. These results represent a call for action to develop and validate new prediction equations in diverse groups of athletes practicing specific disciplines, or groups of athletes with similar body composition characteristics based on the sport modality (e.g., team sports, endurance, or velocity/power).

Despite the encouraging results of this study, some limitations merit consideration. First, due to the wide range of generalized and athletic predictive equations, we had to randomly choose a maximum of two for each group. However, we strictly selected all the predictive equations based on the highest methodological standards. Secondly, when comparing the anthropometry-based with the DXA-derived prediction equations, for three of the selected equations, we had to apply a further equation [53] to convert body density to FM%. Lastly, the present findings cannot be generalized to other sports disciplines, and they cannot be extended to BIA measurements obtained from different technologies or sampling frequencies.

This study presents novel perspectives for the comparison between BIA, anthropometry, and a reference method for the prediction of FM with different modalities of predictive equations. This prompts future research focusing on specificity in procedures when validating double indirect tools for assessing body composition. As such, referring only to generalized equations may result in inaccurate estimations. This is not surprising, given that recent studies used a limited group of equations when comparing BIA and anthropometry [12,14]. Furthermore, there is now a wide range of available commercial BIA devices, used in research articles, that do not provide information on the equation used for measuring FM% in athletes [9]. Therefore, caution should be applied when interpreting data extracted from generalized equations or technologies. In addition, further studies that include athletes, exercisers, and non-athletes should be considered, and the sensitivity of anthropometric or bioimpedance measures should be studied as pre-screening indices, establishing specific parameters that aid in the choice of the prediction equation.

## 5. Conclusions

The use of sport-specific predictive equations resulted in valid FM% estimation, regardless of the BIA or anthropometry use. Although the FM% predicted with the athletic equations did not differ from DXA-derived predictions, lower accuracy was found when compared to sport-specific equations in this cohort of futsal players. Generalized BIA-based predictive equations overestimated FM%, whereas anthropometry-based predictive equations underestimated FM%. In conclusion, BIA and anthropometry can be used interchangeably, allowing for valid FM% estimations, provided that sport-specific equations are applied.

## Figures and Tables

**Table 1:** Characteristics of the selected BIA- and anthropometry-based predictive equations for fat mass estimation.

Authors	Equation	Sample	Methodology	Note
**BIA-Based Predictive Equations**				
Generalized equations				
Lukaski and Bolonchuk [21]	(1) FFM (kg) = 0.734 × (S^2^/R) + 0.116 × Wt + 0.096 × Xc + 0.876 × gender – 4.03 (2) FM% = (Wt − FFM)/Wt × 100	114 men and women	Foot-to-hand BIA at 50 kHz vs. underwater weighing	gender coded as 0 = female, and 1 = male
Sun et al. [22]	(1) FFM (kg) = −10.68 + 0.65 × (S^2^/R) + 0.26 × Wt + 0.02 × R (2) FM% = (Wt − FFM)/Wt × 100	1474 men and women	Foot-to-hand BIA at 50 kHz vs. underwater weighing	
Athletic equations				
Matias et al. [25]	(1) FFM (kg) = −2.261 + 0.327 × (S^2^/R) + 0.525 × Wt + 5.462 × gender (2) FM% = (Wt − FFM)/Wt × 100	142 male and female athletes of different sports (basketball, handball, combat sports, pentathlon, rugby, soccer, swimming, track and field athletic sports, triathlon, volleyball, tennis, and sailing)	Foot-to-hand BIA at 50 kHz vs. 4C modeling	gender coded as 0 = female, and 1 = male
Stewart et al. [26]	(1) FM (g) = 429.4 × Wt − 283.6 × (S^2^/R) − 73.1 × Xc − 134.1 (2) FM% = (FM_g_/1000)/Wt × 100	82 male athletes of different sports (cycling, racket sports, rowing, rugby, running, strength sports, and triathlon)	Foot-to-hand BIA at 50 kHz vs. DXA	
Sport-specific equations				
Matias et al. [29]	(1) FFM (kg) = −8.865 + 0.437 × Wt + 0.186 × Xc + 0.415 × (S^2^/R)	66 male elite futsal players	Foot-to-hand BIA at 50 kHz vs. DXA	
**Anthropometry-based predictive equations**				
Generalized equations				
Durnin and Womersley [23]	(1) BD (g/cm^3^) = 1.16 − 0.06 × ((LOG(4SKF)) (2) FM% = 495/BD − 450 (Siri’s formula)	481 men and women	Manual anthropometry vs. underwater weighing	4SKF = sum of biceps, triceps, subscapular, and iliac skinfolds
Lean et al. [24]	(1) BD (g/cm^3^) = 1.1862 − (0.0684 × LOG(4SKF) − (0.000601 × age) (2) FM% = 495/BD − 450 (Siri’s formula)	147 men and women	Manual anthropometry vs. underwater weighing	4SKF = sum of biceps, triceps, subscapular, and iliac skinfolds
Athletic equation				
Evans et al. [27]	FM% = 8.997 + 0.24658 × (3SKF) − 6.343 × (gender) − 1.998 × (race)	132 male and female athletes of different sports (football, basketball, volleyball, gymnastics, swimming, and track, and field)	Manual anthropometry vs. 4C modeling	3SKF = sum of abdomen, mid-thigh, and triceps skinfolds; gender coded as 0 = female, 1 = male and race coded as 0 = white, 1 = black
Withers et al. [28]	(1) BD (g/cm^3^) = 1.0988 − (0.0004 × 7SKF) (2) FM% = 495/BD − 450 (Siri’s formula)	207 male athletes of different sports (badminton, basketball, cycling, field hockey, field lacrosse, gymnastics, speed roller skating, squash, swimming, and volleyball)	Manual anthropometry vs. underwater weighing	7SKF = sum of biceps, triceps, subscapular, supraspinal, abdominal, mid-thigh, and calf skinfolds
Sport-specific equation				
Giro et al. [30]	FM% = −0.620 + 0.159 × 4SKF + 0.120 × waist circumference (cm)	78 male elite futsal players	Manual anthropometry vs. DXA	4SKF = sum of triceps, abdomen, iliac crest, and mid-thigh skinfolds

Abbreviations: FFM, fat-free mass; FM, fat mass; BD, body density; LOG10, logarithm to base 10; BIA, bioelectrical impedance analysis; 4C, four-compartmental model; DXA, dual-energy X-ray absorptiometry; S, Stature (cm); R, resistance (ohm); Xc, reactance (ohm); Wt, body mass (kg).

**Table 2:** Validation of the selected BIA- and anthropometry-based predictive equations in the futsal players.

		Regression Analysis		CCC analysis			Agreement Analysis		
	Mean ± SD	r^2^	SEE (kg)	CCC	ρ	C_b_	Bias	95% LoA	Trend
FM%_DXA_	15.6 ± 3.6	-	-	-	-	-	-	-	-
**BIA-based predictive equations**									
Generalized equations									
Lukaski and Bolonchuk [21]	18.3 ± 5.3 *	0.53	2.46	0.641	0.880	0.728	2.69	−4.5; 9.9	r = 0.406; *p* < 0.001
Sun et al. [22]	16.8 ± 3.9 *	0.57	2.33	0.719	0.757	0.951	1.13	−4.0; 6.3	r = 0.034; *p* = 0.790
Athletic equations									
Matias et al. [25]	15.2 ± 5.1	0.69	1.99	0.774	0.829	0.933	−0.48	−6.2; 5.2	r = 0.451; *p* < 0.001
Stewart et al. [26]	14.8 ± 4.5	0.53	2.44	0.682	0.729	0.936	−0.80	−7.3; 5.6	r = 0.287; *p* = 0.020
Sport-specific equations									
Matias et al. [29]	15.2 ± 3.2	0.64	2.12	0.799	0.804	0.994	−0.30	−4.5; 3.9	r = −0.217; *p* = 0.083
**Anthropometry-based predictive equations**									
Generalized equations									
Durnin and Womersley [23]	13.6 ± 3.5 *	0.62	2.19	0.670	0.786	0.853	−2.04	−7.3; 5.6	r = −0.138; *p* = 0.271
Lean et al. [24]	13.9 ± 4.1 *	0.64	2.12	0.716	0.800	0.895	−1.72	−6.6; 3.1	r = 0.357; *p* = 0.003
Athletic equations									
Evans et al. [27]	14.9 ± 5.1	0.70	1.93	0.774	0.838	0.925	−0.67	−6.3; 5.0	r = 0.646; *p* < 0.001
Withers et al. [28]	15.1 ± 3.9	0.62	2.16	0.778	0.792	0.932	−0.55	−5.3; 4.2	r = 0.279; *p* = 0.023
Sport-specific equations									
Giro et al. [30]	15.9 ± 3.2	0.81	1.58	0.890	0.900	0.988	0.33	−2.7; 3.4	r = 0.235; *p* = 0.057

Note: r^2^, coefficient of determination; SEE, standard error of estimation; CCC, concordance correlation coefficient; ρ, precision; Cb, accuracy; LoA, limits of agreement; r, coefficient of correlation; BIA, bioelectrical impedance analysis; DXA, dual-energy x-ray absorptiometry. * = Significant differences from DXA (*p* < 0.05).

## Data Availability

Data generated and analyzed during the current study are available from the corresponding author upon reasonable request.
